# The Interplay between Chronotype and Emotion Regulation in the Recognition of Facial Expressions of Emotion

**DOI:** 10.3390/bs13010038

**Published:** 2022-12-31

**Authors:** Isabel M. Santos, Pedro Bem-Haja, André Silva, Catarina Rosa, Diâner F. Queiroz, Miguel F. Alves, Talles Barroso, Luíza Cerri, Carlos F. Silva

**Affiliations:** 1 William James Center for Research, University of Aveiro, 3810-193 Aveiro, Portugal; 2 Department of Education and Psychology, University of Aveiro, 3810-193 Aveiro, Portugal; 3 CINTESIS@RISE, University of Aveiro, 3810-193 Aveiro, Portugal; 4 ISEIT—Instituto de Estudos Interculturais e Transdisciplinares, Piaget Institute, 2805-059 Almada, Portugal

**Keywords:** chronotype, morningness, eveningness, emotion regulation, expressive suppression, cognitive reappraisal, facial expressions, emotion recognition

## Abstract

Emotion regulation strategies affect the experience and processing of emotions and emotional stimuli. Chronotype has also been shown to influence the processing of emotional stimuli, with late chronotypes showing a bias towards better processing of negative stimuli. Additionally, greater eveningness has been associated with increased difficulties in emotion regulation and preferential use of expressive suppression strategies. Therefore, the present study aimed to understand the interplay between chronotype and emotion regulation on the recognition of dynamic facial expressions of emotion. To that end, 287 participants answered self-report measures and performed an online facial emotion recognition task from short video clips where a neutral face gradually morphed into a full-emotion expression (one of the six basic emotions). Participants should press the spacebar to stop each video as soon as they could recognize the emotional expression, and then identify it from six provided labels/emotions. Greater eveningness was associated with shorter response times (RT) in the identification of sadness, disgust and happiness. Higher scores of expressive suppression were associated with longer RT in identifying sadness, disgust, anger and surprise. Expressive suppression significantly moderated the relationship between chronotype and the recognition of sadness and anger, with chronotype being a significant predictor of emotion recognition times only at higher levels of expressive suppression. No significant effects were observed for cognitive reappraisal. These results are consistent with a negative bias in emotion processing in late chronotypes and increased difficulty in anger and sadness recognition for expressive suppressor morning-types.

## 1. Introduction

Emotion processing and recognition assume a prominent role in decision making within survival-relevant behavior, and were extremely important in overcoming the challenging and throbbing paths of evolution [1]. Recognizing the emotions expressed by the face is crucial to modulate our social interactions. Faces allow the extraction of an extensive range of information, which translates into signals of threat, danger, empathy, intention of prosocial or affiliative behavior, among others (e.g., [2,3]). The ability to recognize the six basic emotions from faces (see [4]) appears very early in life and is shaped by a large number of factors, namely by individual differences (e.g., [5,6,7,8]). One of the individual differences dimension that has been shown to have a noteworthy influence on emotional face processing ability is chronotype (e.g., [9,10]).

### 1.1. Chronotype and Emotional Face Recognition

Chronotype can be conceptualized as an individual’s preference for morning/evening activities [11] but also as a circadian rhythmicity predisposition, that interacts, for example, with temporal variations in sleep patterns, core body temperature or hormone levels [12]. According to this preference or individual predisposition, people can be grouped into either morning-types, evening-types or those in between called intermediate-types [13]. Morning-types prefer doing challenging activities in the morning while evening-types feel at their best later in the day (see [14]). Intermediate-types can oscillate between having characteristics similar to morning-types and to evening-types, or having neither.

Regarding the interplay between chronotype and emotion recognition from faces, one of the most salient results from the literature shows that evening-types have an increased recognition ability for sad facial expressions (e.g., [15]). Interestingly this pattern was maintained independently of sleep quality, mood, age and gender [10], indicating a negative bias in emotional processing in evening chronotypes. This result is consistent with studies showing a negative relationship between positive affect and eveningness [16] and with those showing that evening-types have more depressive symptoms (see [17]), more nightmares [18], or a higher prevalence of seasonal affective disorders [19], depression, and anxiety [20]. In addition to depressive bias, several studies show that evening-types present higher levels of impulsivity and irritability and tend to have more expressions of anger [20,21].

Studies that have tried to explain/understand the negative bias for evening-types have focused on genetic, neural and chronobiological factors. A study exploring the neural correlates of this negative bias showed an increased amygdalin response to fearful vs. happy faces in evening-types, indicating a greater emotional reactivity to negative valence stimuli [22]. Additionally, some studies have shown that the association between evening-types and mental illness has been justified by the attenuated neural response to reward in these individuals (see [23]). Regarding genetic factors, some studies have shown that chronotype and depression seem to be both heritable and share some underlying genetic variance, which may explain the negative association between eveningness and mental health (e.g., [24]). Additionally, Lane et al. [25] found that loci linked with chronotype were overrepresented in genes related with fear and behavioral defense responses. From a chronobiological point of view, the most reported explanation is related to the fact that evening-types have stronger misalignment between endogenous circadian timing and the timing of social commitments, leading to a higher prevalence of mental health disorders (e.g., [26].)

### 1.2. Chronotype, Emotion Regulation Strategy and Emotional Processing

James Gross [27] defined emotion regulation as the set of strategies or processes through which individuals manage their emotions, and the way they are experienced and expressed. Additionally, according to Gross [27,28], emotion regulation processes can be divided into two major strategies, the antecedent-focused emotion regulation strategy, also called Cognitive Reappraisal, and the response-focused emotion regulation strategy, also defined as Expressive Suppression. Cognitive reappraisal can be conceptualized as the attempt to reappraise an emotion-eliciting situation in order to modify its meaning and change its emotional impact in the present, but also in the future (e.g., [29]). On the other hand, expressive suppression is defined as the inhibition of the emotion expressing behavior towards a certain situation (e.g., [29]). Emotion regulation research has highlighted a major role of this ability underlying many forms of psychopathology [30]. In addition, emotion regulation strategies modulate the pattern of neuronal activity in emotion-related brain areas, such as the amygdala, thalamus and midbrain, in response to negative stimuli [31] and emotion regulation difficulties have been associated with lowered emotional recognition ability (e.g., [32,33]).

In recent years, some studies have focused on the relationship between chronotype and emotion regulation strategies. Specifically, a study conducted by Watts and Norbury [34] showed a positive association between the use of expressive suppression and eveningness, and a positive association between morningness and the use of cognitive reappraisal strategies. A similar result was found by Antúnez [35], where morning-types showed higher values of cognitive reappraisal. This study also shows that evening-types with the least propensity to reappraise have a higher tendency to maintain maladaptive beliefs highly related to psychological well-being and psychopathology [35].

There is evidence of a positive correlation between the recognition of facial expressions of emotion and emotion regulation [36] and studies also showed that the processing of emotional faces seems to depend in part on the emotion regulation strategy [37]. Moreover, attentional bias to emotional faces predicted emotion regulation difficulties (e.g., [38]), and expressive suppression significantly moderated the relation between depressive symptoms and the recognition of facial expressions of anger [39]. Thus, considering the effect of emotion regulation strategies on the processing of emotional stimuli, and the relationship between eveningness and expressive suppression, is it possible that emotion regulation strategies will influence the association between chronotype and emotional processing?

Emotion regulation strategies are significantly associated with various aspects of mental health [40,41]. Additionally, emotion regulation difficulties seem to be a significant factor in the development, maintenance and treatment of a diverse range of psychopathologies (e.g., [42]), with a particular relevance on depression [43,44]. As previously highlighted, circadian preference has also been related to certain mental health difficulties [17,23,26], as well as with a biased processing of emotional stimuli [9,10,15]. Therefore, this knowledge provided by this study will contribute to a better understanding of the mechanisms underlying the relationship between circadian preferences and emotional processing, and how emotion regulation can play a role in modulating these mechanisms. This insight can have important implications for the comprehension and intervention with clinical cases.

### 1.3. The Present Study

The aim of the present study was to understand the interplay between chronotype, emotion regulation and emotion processing of faces, namely assessing whether emotion regulation was a significant moderator of the relationship between morningness-eveningness and the recognition of dynamic facial expressions of emotion.

One of the assets of this study was the type of stimuli and task used to investigate emotion recognition from facial expressions. The literature has suggested that emotion recognition from dynamic stimuli is facilitated, in comparison to static images, and this benefit is more evident for subtle facial expressions [45,46]. This can be due to differences in the neural pathway for processing the two types of stimuli, to an increased sensitivity to changes in the dynamic stimuli, or to the temporal characteristics of the unfolding expression [47]. The use of dynamic videoclips and the analysis of response times (RTs) has been suggested as a more adequate method to assess emotion recognition [48]. Additionally, RTs should be measured towards a single response option, and not when there is a high number of options, such as when a decision has to be made among six basic emotion labels [48]. Taking these findings in consideration, we opted to use dynamic videoclips, with the facial expressions changing gradually over time from a neutral facial expression to a full emotional expression. Additionally, in order to have a more precise measure of emotion recognition [48], RTs were registered and analyzed considering the moment of the videoclips where participants were able to identify an emotional expression and stopped the video. Only subsequently participants were required to indicate the label for the identified emotion, among six possible options.

To reach our aims, an online study was developed, where participants completed self-report measures of chronotype and emotion regulation, and performed a facial expression recognition task from short video clips where a neutral expression progressively morphed into one of the full basic emotional expressions (happiness, sadness, fear, disgust, anger and surprise). Participants were asked to stop the videoclip as soon as they could recognize the facial expression, and they should subsequently identify it. Data analysis examined the relation between chronotype and emotion recognition, and how the emotion regulation strategies (expressive suppression and cognitive reappraisal) moderated that relation. Considering the influence of time-of-day (ToD) on chronotype effects (see [14]), the moderating effect was also tested when statistically controlling for ToD.

Although associations have been established between chronotype and the processing of emotional stimuli, as well as between chronotype and emotion regulation strategies, the moderating role of emotion regulation on the relation between morningness-eveningness and the processing of facial expressions of emotion has not yet been explored, to the best of our knowledge. Additionally, the use of dynamic facial expression stimuli will allow to explore changes in sensitivity to the unfolding of the emotional expression signals and in what way the emotion regulation strategy may moderate how morningness-eveningness preference modulates this ability.

## 2. Method

### 2.1. Participants and Procedure

Volunteers were recruited to participate in this online study through social media and institutional mailing lists. They accessed the experiment through a shared link, which directed them to a questionnaire implemented in LimeSurvey, including a few sociodemographic questions and other self-report measures described below. The last page of the questionnaire contained two links, which directed participants to a Pavlovia webpage—https://pavlovia.org/ [49], the only difference between them being task counterbalancing. The study protocol included two experimental tasks, with only one of them being relevant to the present study. The experimental tasks were built in PsychoPy for Windows (Version 2021.1.4, [50], and uploaded to Pavlovia for online data collection).

From 914 volunteers who accessed the shared LimeSurvey link, only 287 participants successfully completed the entire questionnaire protocol and the facial emotion recognition task. Participants (81.2% females) were aged between 18 and 59 years old (Mean = 28.4, SD = 10.0). Before starting the study protocol, participants completed an informed consent online. This study was approved by the Ethics and Deontology Committee of the University of Aveiro (Nº40-CED/2019, 22 January 2020).

### 2.2. Materials

#### 2.2.1. Chronotype

The reduced version of the Morningness-Eveningness Questionnaire (rMEQ: [51]; European Portuguese adaptation by [52]) was used to assess participants’ chronotype. The questionnaire consists of five multiple-choice items and shows good psychometric properties (Loureiro and Garcia-Marques, 2015). Total scores range from 4 to 25 and have cut-off points which classify individuals into evening-types (scores between 4 and 11), neutral-types (scores between 12 and 17) or morning-types (scores of 18–25).

#### 2.2.2. Emotion Regulation

Emotion regulation was assessed through the Emotion Regulation Questionnaire (ERQ) [53,54]. This is a self-report questionnaire which includes 10 items, assessed on a 7-point Likert scale, varying from 1 = “Totally disagree” to 7 = “Totally agree”. It provides scores on two dimensions of emotion regulation, namely “cognitive reappraisal” (6 items) and “expressive suppression” (4 items), and shows good psychometric properties [54]. High scores on each of the two subscales represent a higher tendency to use that particular emotion regulation strategy.

#### 2.2.3. Facial Emotion Recognition Task

To evaluate the recognition of facial expressions of emotion, we used a dynamic task with videoclips in which the face of a person gradually morphed from a neutral expression into the maximum expression of one of the six basic emotions (anger, disgust, fear, happiness, sadness, and surprise). The faces that were used to create the videoclips were selected from the Karolinska Directed Emotional Faces System (KDEF) database [55]. The photos of six different individuals (half male, half female) were selected, each displaying the six basic emotions and a neutral expression. The software Abrosoft FantaMorph—version 5 was used to delineate all the images, using 800 points in each face, located on the main features (the limits of the face, eyes, mouth, nose, eyebrows, ears, and other surface features). Following this procedure, 100 intermediate images between the neutral expression and each full expression for each individual were created. These individual images were then used to create animated videoclips for each individual/emotion (in a total of 36 videoclips), with a duration of 15 s, which was the time it took for each face to completely change from neutral to the full expression of an emotion. Four additional videoclips of different individuals (created in the same way) were used for practice trials.

**Figure 1:**
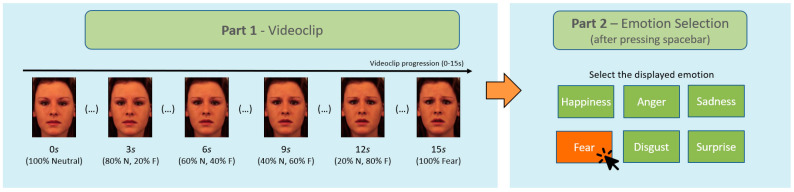
Trial scheme of the facial emotion recognition Task.

Each trial initiated with a fixation cross presented for 1 s, after which the videoclip started. Participants were asked to press the spacebar as soon as they were able to determine which emotion was presented. If they did not press the spacebar, the video would run until the end (15 s). In both cases, a new screen appeared asking participants which emotion they were able to identify from the previously presented clip. Six rectangular boxes appeared on the screen, labelled with each of the basic emotions, and participants were required to indicate with a left button mouse click the identified emotion (Figure 1). The task consisted of four practice trials and 36 test trials, randomly presented.

### 2.3. Data Analysis

Due to technical problems, we discarded 0.53% of trials, which corresponded to instances where RTs—time elapsed between the onset of the stimuli and participants’ spacebar keypress—exceeded the duration of the stimuli (this is, when the recorded time was longer than the maximum duration of 15 s). Furthermore, we applied a filter to our data, so that trials whose RTs were lower than two standard deviations of the mean were also discarded. This further eliminated 2.04% of trials. The upper limit corresponded to stimuli durations, as participants could watch each animation until it disappeared from the screen (15 s after onset). Considering that the RTs for sadness, happiness, surprise and disgust did not follow a normal distribution, square root transformations were performed on the data.

Considering the aim of the study, general linear models (GLMs) fitted with ordinary least-squares (OLS) were performed. To test whether expressive suppression moderated the relationship between chronotype and RTs in recognizing emotional facial expression, models with Equation (1) were performed for each emotion.

RT = 1 + rMEQ + ERQ_ES + rMEQ:ERQ_ES(1)

To the models of emotions that showed a significant interaction (rMEQ:ERQES) the variable ToD was subsequently included. The tested model complies with Equation (2).

RT = 1 + rMEQ + ERQ_ES + ToD + rMEQ:ERQ_ES(2)

Considering the long-standing evidence of the interaction between chronotype and ToD in cognitive tasks (Schmidt, 2007), this last model (Equation (2)) aimed to control the ToD variance and verify if the previously obtained interaction was maintained. In the two equations above, RT corresponds to the response times in correctly recognizing the emotional facial expression, rMEQ is the measure of chronotype, and the ERQ_ES is the measure of expressive suppression. The ToD is the time after midnight when participants performed the task.

To test whether cognitive reappraisal moderated the relationship between chronotype and RTs in recognizing emotional facial expression, models with Equation (3) were performed for each emotion.

RT = 1 + rMEQ + ERQ_CR + rMEQ:ERQ_CR(3)

As no interaction effects (rMEQ:ERQCR) were observed, no further analyses were performed for cognitive reappraisal.

In Equation (3), ERQ_CR is the measure of cognitive reappraisal. All other elements of the equation are the same as above (Equation (1)).

Statistical analyses were conducted in Jamovi (Version 2.2.5.0, The jamovi project, 2021).

## 3. Results

The statistical models’ results are reported separately for each emotion regulation strategy.

### 3.1. Models with Expressive Suppression

An overview of the models’ results can be seen in Table 1. Main effects of rMEQ were obtained for happiness [*F*(1, 252) = 4.410, *p* = 0.037, *η*^2^*_p_* = 0.017], sadness [*F*(1, 253) = 4.223, *p* = 0.041, *η*^2^*_p_* = 0.017] and disgust [*F*(1, 250) = 5.278, *p* = 0.022, *η*^2^*_p_* = 0.021] and a marginal effect was obtained for surprise [*F*(1, 253) = 3.770, *p* = 0.053, *η*^2^*_p_* = 0.015]. These results showed that higher eveningness was associated with shorter RTs in the identification of happiness, sadness and disgust. Additionally, main effects of expressive suppression were found for sadness [*F*(1, 253) = 7.159, *p* = 0.008, *η*^2^*_p_* = 0.028], disgust [*F*(1, 250) = 4.138, *p* = 0.043, *η*^2^*_p_* = 0.016], anger [*F*(1, 254) = 7.618, *p* = 0.006, *η*^2^*_p_* = 0.029] and surprise [*F*(1, 253) = 6.149, *p* = 0.014, *η*^2^*_p_* = 0.024]. Higher expressive suppression values were positively associated with longer RTs in identifying sadness, disgust, anger and surprise from faces. Remarkably, an interaction between rMEQ and expressive suppression was obtained for sadness [*F*(1, 253) = 4.330, *p* = 0.038, *η*^2^*_p_* = 0.017] and for anger [*F*(1, 254) = 5.247, *p* = 0.023, *η*^2^*_p_* = 0.020].

**Figure 2:**
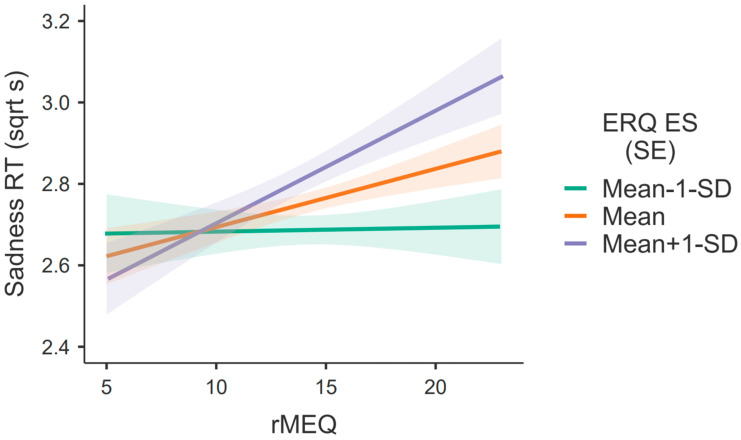
Simple main effects of chronotype on response times (RT) in identifying sadness from faces according to the expressive suppression levels (Mean ± 1 SD); RT was squared root transformed due to normality issues. (SE) = standard error of the mean—shaded zone surrounding the lines.

To further explore these interactions, simple main effects of rMEQ were performed using expressive suppression as a moderator. Regarding sadness, the results show that the effect of rMEQ on RT only exists at average [Mean; F(1, 250) = 4.223, *p* = 0.041, *η*^2^*_p_* = 0.017] or high levels [Mean + 1 SD; F(1, 250) = 8.992, *p* = 0.003, *η*^2^*_p_* = 0.035] of expressive suppression (see Figure 2).

**Figure 3:**
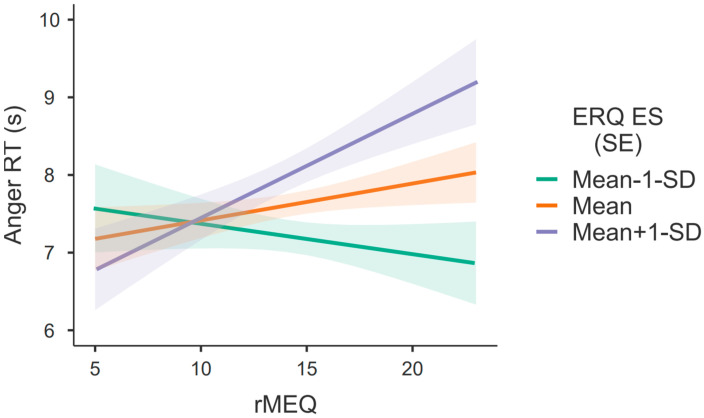
Simple main effects of chronotype on response times (RT) in identifying anger from faces, according to the expressive suppression levels (Mean ± 1 SD). (SE) = standard error of the mean—shaded zone surrounding the lines.

Similar results were obtained for anger, but in this case the effect of rMEQ is only significant at high levels [Mean + 1 SD; F(1, 251) = 5.960, *p* = 0.015, *η*^2^*_p_* = 0.023] of expressive suppression (see Figure 3). Altogether, the obtained results show that the relationship between rMEQ and RT for sad and angry faces was moderated by the level of expressive suppression.

The absence of a main effect of rMEQ for anger (Table 1) seems to be explained by the negative relationship (although non-significant) between rMEQ and RT for angry faces, when expressive suppression levels are low (see Figure 3), which counters the overall effect observed for the other two levels of expressive suppression.

Conceptually, and looking at the graphs in Figure 2 and Figure 3, a superimposition of the lines on the eveningness extremes (lower values of rMEQ) and a clear separation of the lines on the morningness extreme (higher values of rMEQ) can be observed. In fact, the main differences are found on the right side of the graphs, i.e., for higher rMEQ values. Regarding rMEQ cut-offs [52], evening-types obtain scores between 4 and 11 and morning-types obtain scores between 18–25. So, it seems that it is the morning-types who are mostly driving the effect, whereby morning types with high expressive suppression are slower to identify sad and angry faces.

Considering the reported importance of ToD for chronotype effects, this variable was added to the model. The results show that, in addition to the moderating effect not disappearing, the effect size of the interaction was slightly improved for both sadness [F(1, 253) = 4.477, *p* = 0.036, *η*^2^*_p_* = 0.018] and anger [F(1, 254) = 5.463, *p* = 0.020, *η*^2^*_p_* = 0.021]. Furthermore, no main effects of ToD were recorded for either emotion, nor was there any change in the main effects of rMEQ achieved in the models without ToD.

### 3.2. Models with Cognitive Reappraisal

An overview of the models’ results can be seen in Table 2. Importantly, as it happened for the expressive suppression models, a main effect of rMEQ was observed for happiness [F(1, 252) = 4.546, *p* = 0.034, *η*^2^*_p_* = 0.018], sadness [F(1, 253) = 4.399, *p* = 0.037, *η*^2^*_p_* = 0.017], and disgust [F(1, 250) = 4.829, *p* = 0.029, *η*^2^*_p_* = 0.019]. These results showed that eveningness was negatively associated with RT in the identification of happiness, sadness and disgust. Contrary to what happened for expressive suppression, in the models with cognitive reappraisal, no significant interactions were observed, suggesting that this emotion regulation strategy does not moderate the relationship between chronotype and RTs in the recognition of the basic emotion expressions. Furthermore, also differing from what occurred to expressive suppression, the main effect of cognitive reappraisal was not significant for any emotion (Table 2).

## 4. Discussion

Recognition of emotions expressed by the face is essential for adaptive social interaction [2]. However, the literature has shown that the ability to identify these expressions depends on individual differences, namely chronotype [10]. One of the most relevant results associates eveningness with a negative bias towards facilitated processing of negative affect faces such as sad facial expressions (e.g., [15]). However, studies have shown that the emotion regulation strategy favored by the individual also has an impact on the emotional processing of faces (e.g., [37]), and that there is a positive association between the use of expressive suppression and eveningness [34]. The aim of the present study was to understand the interplay between chronotype, emotion regulation strategies and emotion processing from faces, namely evaluating whether emotion regulation was a significant moderator of the relationship between morningness-eveningness preference and the recognition of dynamic facial expressions of emotion. The use of dynamic stimuli, together with the assessment of RTs in emotion recognition, represent an adequate and sensitive method to assess facial expression recognition [48].

### 4.1. Chronotype and Emotional Facial Expressions

The results of the present study showed that eveningness was negatively associated with RT in the identification of happiness, sadness and disgust, that is, evening-types were significantly faster to identify those facial expressions. Regarding sadness, this result was as expected, since several studies had already shown that evening-types have an increased recognition ability for sad facial expressions (e.g., [10,15]). These results are supported by the positive relationship between eveningness and depression (see [56] for a review), and the fact that individuals with depression show preferential processing of negative material (see [57] for a review), namely better performance for sad faces [58]. The literature has suggested that the negativity bias in late chronotypes may be related to higher cognitive reactivity, especially increased rumination [59] or by the weaker stability of affect that characterizes evening-types [60]. Additionally, a study by Carciofo [61] found increased negative affect among evening types. This instability in affect, as well as this negative bias, may also be related to a stronger misalignment between endogenous circadian timing and the timing of social commitments [26]. Recent research proposes that low positive affect may mediate the positive association between eveningness and depression [62], and interestingly, as with chronotype, positive affect obeys an endogenous circadian rhythm and is controlled by both circadian and homeostatic components [63]. This literature can also help explain the result obtained for disgust, since disgust is also considered a negative emotion [64], and enhanced neural sensitivity to facial expressions of disgust have been found in association with depression [65]. Therefore, the present result can also be interpreted in consonance with the negativity bias commonly found for late chronotypes [10].

The association between eveningness and faster RTs in identifying happiness; however, was contrary to our expectations and seems incoherent with the reported literature. Although no immediate or definitive explanation for this result can be advanced at the present moment, a possibility is that this result could be due to an interplay between individual characteristics of late chronotypes and specificities of the task employed in this study. Evening-type individuals show higher levels of impulsivity and lower levels of response inhibition [66] and the cognitive factor impulsivity/inhibition is significantly associated with both implicit emotion recognition speed and explicit emotion identification speed [67]. On the other hand, happiness is typically the easiest and fastest emotion to be recognized, and requires shorter stimulus exposure times [64,68]. Thus, conjugating the characteristics of the present task (dynamic facial expression detection and identification), the easier overall recognition of happy facial expressions and the higher impulsivity of late chronotypes, it is possible that these factors might explain why eveningness was associated with faster responses for a positive facial expression. Nonetheless, this is only a tentative explanation and more studies are needed to replicate and disentangle this effect.

### 4.2. Emotion Regulation and Emotional Facial Expressions

The results of the present study showed that higher expressive suppression scores were associated with longer RTs in the recognition of sadness, disgust, anger and surprise from faces. These results were expected considering previous studies that show that participants who suppressed their emotions were slower to recognize facial expressions [69]. Regarding negative emotions (sadness, disgust and anger), the literature has also shown that when participants use suppression, they are slower to respond during unpleasant stimuli [70]. Furthermore, there seems to be a correlation between suppression and cognitive control for negative information [71]. With regard to surprise, this effect can be explained by the ambiguity of this facial expression, particularly in a dynamic task. Indeed, surprised facial expressions can signal both pleasant and unpleasant outcomes [72,73]. 

Despite the reported results for expressive suppression, no effects of cognitive reappraisal were obtained. This result was not entirely expected considering evidence that higher cognitive reappraisal scores were correlated with faster RTs in response inhibition to sad faces [37]. The lack of significant effects might be related to the nature of the task, namely the free viewing setting with the sole instruction of identifying the facial expression and the absence of an instruction to use a certain emotion regulation strategy. In fact, a cognitive reappraisal strategy requires the individuals to reconceptualize emotion-inducing events and involves additional resources for cognitive reconstruction [74], which might not take place or be triggered by the performed task.

More studies are needed to understand the actual role of cognitive reappraisal in the processing of facial expressions of emotion, as well as clarify the role of expressive suppression in the emotional processing of surprise, namely through neurobiological correlates.

### 4.3. Moderating Role of Emotion Regulation on the Relationship between Chronotype and Facial Expression Recognition

In the present study, expressive suppression was shown to moderate the relationship between chronotype and emotional processing of sadness and anger faces. Regarding sadness, the relationship between chronotype and the time taken to identify this emotion was only significant in individuals who scored average and high in expressive suppression. For anger, the effect of chronotype was only significant for high scores of expressive suppression. These results suggest that morning-types with high expressive suppression are slower to identify sad and angry faces. Although early chronotypes tend to score higher on cognitive reappraisal (see [35]), it seems that when they are high expressive suppressors they have increased difficulty in processing sadness and anger from faces. This result is consistent with previous results showing that expressive suppression leads to slower responses to negative stimuli (e.g., [70]), which were also verified in our study. Interestingly, for lower scores of expressive suppression, there was no association between chronotype and RT in the recognition of sad and angry faces.

Conceptually, considering the unidimensional structure of chronotype underlying the used questionnaire, we could also interpret that high expressive suppression facilitates the emotional processing of evening-types. However, this interpretation seems more implausible, as it would imply that expressive suppression, which has a detrimental effect on the processing of negative stimuli [69], facilitated the recognition of negative emotions in evening-types. Even so, a possible explanation would be the existence of a negative bias in evening-types, which makes them more adept at processing negative stimuli, especially at higher levels of expressive suppression. In fact, both negative affect [61] and expressive suppression [35] are related to eveningness. Therefore, both mechanisms might simultaneously contribute to the observed results, although the graphic display of the moderating effects favors a stronger influence of expressive suppression levels at the morningness end of the chronotype continuum.

Importantly, the moderation effects remain intact even after the inclusion of ToD in the model. This result strengthens the importance of the moderation effect, considering the known interplay between ToD and chronotype and its effect on performance [14]. These results suggest that the emotion regulation strategy should be a relevant variable to consider in studies that investigate the relationship between chronotype and emotional processing, namely those that explore negative bias.

### 4.4. Limitations and Future Studies

There are some limitations that can be pointed out to the present study, which should be considered in future studies. The first is related to the sample that, despite the wide age distribution, has a large proportion of university students. Young adults, especially students, have particular social rhythms, with many late activities and a usually active nigh life, which can interact with biological rhythms, namely with chronotype and its expression. Furthermore, the increased nocturnal social life generally characteristic of university students might minimize the impact of the reduced social contact that is commonly associated with evening-types, and which is one of the advanced explanations for their increased negative affect, although not entirely supported [62]. Thus, future studies should attempt to replicate and extend the present findings with a more age balanced sample.

In addition, there is another more conceptual limitation. The model underlying the chronotype assessment questionnaire used in the present study [51,52] is unidimensional, which prevents the understanding of whether the verified effects are more related, for example, to higher morningness or to lower eveningness. Future studies could use other instruments for assessing morningness–eveningness preference based on multidimension conceptual models, such as the Morningness–eveningness-stability-scale improved (MESSi) [75], which might be better suited to explore differences between the morningness and eveningness dimensions.

## 5. Conclusions

The present study aimed to understand the interplay between chronotype and emotion regulation strategies on the recognition of dynamic facial expressions of emotion. The results obtained indicate faster processing of sadness and disgust in association with higher eveningness, which supports the existence of a negative bias in emotion processing in late chronotypes [10]. A positive association between eveningness and RTs in the recognition of happiness was also observed, which might be explained by an interplay between the characteristics of the task, the easiness of happiness recognition and personal characteristics of late chronotypes, but requires further replication.

Importantly, the present results evidence a significant moderating effect of emotion regulation strategies on the relation between chronotype and facial expression recognition, which remains intact even after controlling ToD effects. Specifically, results are consistent with an increased difficulty in the recognition of sadness and anger in morning-types who score high on expressive suppression, regardless of ToD. No significant moderating effects were observed for cognitive reappraisal. Expressive suppression has been associated with slower responses to negative stimuli [70], and our results indicate that this is particularly evident for morning-types. Thus, it seems that lower emotional expressivity in morning-types is detrimental to the processing of negative stimuli. Nonetheless, a full understanding of this effect requires further investigation. Future studies should attempt to disentangle the impact of emotion regulation strategies considering the dimensions of morningness and eveningness differentially. Additionally, the inclusion of clinical samples would provide a better understanding regarding how these mechanisms might underlie some psychopathological manifestations involving affect dysregulation.

In summary, results highlight the importance of chronotype as a relevant variable in interplay with emotion regulation to better understand emotional functioning. This can have significant implications in terms of case formulation and clinical practice. Considering that the association between alterations in circadian rhythms and the onset of depressive disorders might be explained, at least partially, by biological mechanisms, chronobiological approaches have been suggested as potentially relevant in the prevention and treatment of depressive disorders [17]. The present study provides further support to the complex interaction between circadian preferences and individual characteristics as a relevant mechanism underlying emotional processing.

## Figures and Tables

**Table 1:** Overview of models with expressive suppression.

Emotion	Main Effect rMEQ	Main Effect ES	rMEQ × ES	Moderating Effect of ES?
Happiness	***p* = 0.037,**	*p* = 0.186,	*p* = 0.416,	NO
	***η*^2^*_p_* = 0.017**	*η*^2^*_p_* = 0.007	*η*^2^*_p_* = 0.003	
Sadness	***p* = 0.041,**	***p* = 0.008,**	***p* = 0.038,**	**YES**
	***η*^2^*_p_* = 0.017**	***η*^2^*_p_* = 0.007**	***η*^2^*_p_* = 0.017**	
Fear	*p* = 0.110,	*p* = 0.806,	*p* = 0.749,	NO
	*η*^2^*_p_* = 0.010	*η*^2^*_p_* < 0.001	*η*^2^*_p_* < 0.001	
Disgust	***p* = 0.022,**	***p* = 0.043,**	*p* = 0.478,	NO
	***η*^2^*_p_* = 0.021**	***η*^2^*_p_* = 0.016**	*η*^2^*_p_* = 0.002	
Anger	*p* = 0.249,	***p* = 0.006,**	***p* = 0.023,**	**YES**
	*η*^2^*_p_* = 0.005	***η*^2^*_p_* = 0.029**	***η*^2^*_p_* = 0.020**	
Surprise	*p* = 0.054,	***p* = 0.014,**	*p* = 0.090,	NO
	*η*^2^*_p_* = 0.015	***η*^2^*_p_* = 0.024**	*η*^2^*_p_* = 0.011	

ES = Expressive suppression. Bold indicates statistically significant effects.

**Table 2:** Overview of models with cognitive reappraisal.

Emotion	Main Effect rMEQ	Main Effect CR	rMEQ × CR	Moderating Effect of CR?
Happiness	***p* = 0.034,**	*p* = 0.453,	*p* = 0.255,	NO
	***η*^2^*_p_* = 0.018**	*η*^2^*_p_* = 0.002	*η*^2^*_p_* = 0.005	
Sadness	***p* = 0.037,**	*p* = 0.632,	*p* = 0.208,	NO
	***η*^2^*_p_* = 0.017**	*η*^2^*_p_* = 0.001	*η*^2^*_p_* = 0.006	
Fear	*p* = 0.086,	*p* = 0.626,	*p* = 0.323,	NO
	*η*^2^*_p_* = 0.012	*η*^2^*_p_* = 0.001	*η*^2^*_p_* = 0.004	
Disgust	***p* = 0.029,**	*p* = 0.889,	*p* = 0.823,	NO
	***η*^2^*_p_* = 0.019**	*η*^2^*_p_* < 0.001	*η*^2^*_p_* = 0.002	
Anger	*p* = 0.240,	*p* = 0.658,	*p* = 0.203,	NO
	*η*^2^*_p_* = 0.005	*η*^2^*_p_* = 0.001	*η*^2^*_p_* = 0.006	
Surprise	*p* = 0.070,	*p* = 0.403,	*p* = 0.869,	NO
	*η*^2^*_p_* = 0.013	*η*^2^*_p_* = 0.003	*η*^2^*_p_* < 0.001	

CR = Cognitive reappraisal. Bold indicates statistically significant effects.

## Data Availability

The data that support the findings of this study are available from the corresponding author, (I.M.S.), upon reasonable request.
